# The Walrus and the Carpenter

**Published:** 1912-06-22

**Authors:** 


### THE WALRUS AND THE CARPENTER.

Thoughtful people may be pardoned for finding an analogy between certain medical critics and one of the chief characters in the notorious conspiracy against the oysters so pathetically narrated by Carroll. The Walrus and the Carpenter at least knew it was sand that they had before them; we do not even know what manner of things are the diseases we have to attack. Notwithstanding progress in many directions we know nothing, or next to nothing, concerning the nature of cancer, of gout, of the common cold, of smallpox, and scarlet fever. For years we have been fighting consumption, and even now we are only dimly realising that our methods are probably all wrong, and that we need revision of the whole gamut of nonsense which has been written and taught about this subject. Well may the Carpenters express the doubt which they naturally feel.

But there is a tendency to debar them from finding even that solace. If one of us ventures to suspect the accuracy of statistics, which are statistics only to those who have had no training in the use of large masses of figures; if another dares question the results obtained by some new method evolved in the laboratory, what shall not be meted out to him in the way of scorn and punishment! What professional man will, in a professional gathering of medical men, have the courage to characterise the numerous transplantation "experiments" so popular in certain American research establishments by the epithet that properly belongs to them? Yet it is well sometimes to remember that the Carpenter is among us, as a counterpoise to the cheery optimism of the Walrus. We are apt to "spread the butter much too thick," and we fail wantonly, sometimes, in admiring other things besides the view.

Therefore, Professor Karl Pearson comes as a healthy, stimulating force to the profession. His logical mind and the experience which he has gained in handling masses of complicated data, make him a keen and competent critic—competent, that is to say, to test the common-sense foundations of theories which rely largely on so-called statistics. He has shown us the fallacious methods of argument adopted by the anti-alcohol section. His candid criticism in this direction has done a world of good. Recently, in a letter to the *Times*, he has touched a matter which many feel should be discussed by the expert layman just as much as by the expert doctor—the basis of our treatment of consumption. As he rightly remarks, "it is fairly clear to anyone who approaches the problem from a neutral standpoint, that medical knowledge on the subject is almost complete chaos at present, and that the right policy of the nation is to try and reach bacteriologically, pathologically, and above all statistically, some real knowledge on the subject before spending thousands of pounds on treatment or on segregation, of which no one at present can predict the result." That is exactly the position which the Carpenters take up. They doubt whether mopping, no matter how large the number of maids may be, will be availing until we know where the sand comes from and how it can be finally disposed of. We have had an elaborate report from the Tuberculosis Commission, but it is a report unsupported by statistical evidence. There are grave and growing objections to the sanatorium and dispensary methods of dealing with the problem, and bacteriology and pathology do not seem to give us real facts, although they supply us with many theories, as the recent discussion in the Congress at Rome proves. Perhaps the real solution lies in applying our energies more towards the sociological and statistical aspects of the question. If that be the case, the profession is indebted to Professor Pearson for drawing attention to the fact. But it will be more deeply obliged to him if he will favour us with an epitome of the lines and methods on which, in his opinion, the practitioner can help in elucidating the matter. We look to him to supply this suggestive epitome and to make it known to those of us who are willing and able to do their share of the work if they only knew how to begin.

